# Identification and isolation of spoilage microbes in conventional dark leafy green vegetable juice during cold storage

**DOI:** 10.3389/fmicb.2026.1831321

**Published:** 2026-05-13

**Authors:** Chenxi Guo, Xiaonuo Long, Xiran Li, Luxin Wang

**Affiliations:** Department of Food Science and Technology, University of California, Davis, Davis, CA, United States

**Keywords:** conventional leafy greens, dark leafy greens vegetables, food safety, food spoilage, High-throughput sequence, refrigerated storage

## Abstract

Chard, collard greens, and kale juices as representative dark leafy green vegetable juices (DLGVJs) have gained increasing attention due to their rich nutritional profiles and health-promoting properties. Conventional dark leafy green vegetables (DLGVs) represent the majority of the DLGV market; however, the microbial changes in conventional DLGVJs during refrigerated storage remain poorly understood. This study aimed to characterize the microbial profiles of fresh and spoiled conventional DLGVJs using both culture-dependent and culture-independent methods, alongside monitoring physicochemical changes (pH and color) over 21 days of refrigerated storage. Results showed a general decrease in pH and a shift toward more saturated color in all DLGVJs. Initial aerobic plate counts (APC) in chard, collard greens, and kale juices were 7.71 ± 0.03, 7.57 ± 0.14, and 7.61 ± 0.09 Log CFU/mL, respectively, while fungal populations were 5.85 ± 0.03, 6.01 ± 0.15, and 6.15 ± 0.03 Log CFU/mL. APC showed an overall decrease while fungal populations exhibited an initial increase followed by a decline during refrigerated storage. High-throughput sequencing revealed *Pseudomonas*, *Leuconostoc*, and *Periweissella* as core spoilage-associated bacterial genera, while *Sporobolomyces*, *Alternaria*, and *Symmetrospora* were predominant fungal genera in spoiled conventional DLGVJs. Although microbial compositions showed similarities between conventional and organic DLGVJs, distinct microbial communities were observed, with some taxa uniquely associated with a specific cropping system. The dominant lactic acid bacteria isolated from DLGVJs were identified as *Enterococcus faecium* and *Leuconostoc mesenteroides*. These findings advance our understanding of microbial ecology in DLGVJs and provide a scientific basis for developing targeted interventions to ensure microbial quality and safety, supporting the growth of the DLGVJ market.

## 1 Introduction

Fruit and vegetable juice products represent a significant segment of the beverage industry (USDA, 2024). Dark leafy green vegetables (DLGVs) such as collard greens, kale, and chard are gaining increased attention as primary ingredients for juice production given their rich nutrient profiles and health-promoting properties (Kumar et al., 2020). Dark leafy green vegetable juices (DLGVJs) are economically and nutritionally relevant derivatives of DLGVs (Pollock, 2016; Satheesh et al., 2020), aligning well with consumers’ healthy lifestyle demand and contributing to the economic growth of the DLGV sector. Conventional and organic farming are the two primary cultivation methods for DLGVs. Conventional systems generally achieve higher yields with low production cost than organic system, resulting in greater market availability and affordability for consumers.

Compared with other leafy greens (e.g. Romaine lettuce), the cleaning and sanitizing of DLGVs can be challenging due to their distinctive surface characteristics, including large and more rough surfaces. For example, a washing procedure including a 5 min soaking (w/v ration 1:50) in 80 ppm peracetic acid solution followed with a 15 s rinsing with MilliQ water is needed to achieve a 1.2 Log reduction of native bacterial populations on kale (Guo et al., 2026). DLGVJs exhibit a darker color and have a more neutral pH compared to other juices due to their high contents of chlorophyll, carotenoids, and alkaline minerals (Atkins et al., 2020; Salehi, 2021). These unique properties increase the susceptibility of DLGVJs to microbial spoilage and can even facilitate the prolonged survival of foodborne pathogens (Barth et al., 2009; Guo et al., 2025b). Indeed, when *Salmonella*, pathogenic *Escherichia coli*, and *Listeria monocytogenes* were inoculated into non-acidified chard, collard green, and kale juices, the pathogens persisted for up to 21 days under refrigerated (4 °C) storage with minimal reduction (Guo et al., 2025b). Given the importance of microbes in juice products, Snyder and Worobo (2018) reported that 97.4% of participating juice processors recognized microbial spoilage as having a significant impact on the brand image of fruit and vegetable juice products. Despite the growing popularity of DLGVJs, there remains a limited understanding of their microbial ecology during storage and how this knowledge can be translated into effective food safety and quality management strategies. Collectively, these gaps highlight the need to systematically evaluate and identify the key spoilage microorganisms associated with DLGVJs.

High-throughput sequencing approaches provide powerful tools for characterizing microbial communities in food matrices. When combined with culture-dependent methods (plating), they enable a more comprehensive understanding of microbial taxa, including identification of microbial communities and the relative abundance of bacteria and fungi present in various food products (De Filippis et al., 2018; Kaur et al., 2017; Rantsiou et al., 2005). For example, De Filippis et al. (2018) identified the important role played by acetic acid bacteria (AAB) during the fermentation of kombucha at 20 or 30 °C. While the plating results obtained from Kappeng or Frateur media both indicated the gradually increase of AAB during the 21-day storage regardless of the fermentation temperatures, the culture-independent V1-V3 region sequencing demonstrated the impact of higher temperature on the selection of different *Gluconacetobacter* species, providing more comprehensive picture of the fermentation process. In addition to profiling the total microbial populations, the use of whole-genome sequencing (WGS) provides an accurate tool for characterizing individual isolates obtained from spoiled products at the species and even strain levels (Peng et al., 2023).

The objectives of this study were to (i) characterize the microbial populations and their dynamics in conventional DLGVJs during refrigerated storage using culture-dependent and culture-independent approaches, (ii) monitor major physicochemical changes during spoilage, including pH and color, and (iii) isolate and characterize dominant lactic acid bacteria associated with spoilage from spoiled DLGVJs. Outcomes of this project support the development of evidence-based processing and storage strategies to improve microbial safety and shelf life, enhance laboratory detection and monitoring of microbial communities in complex juice matrices, and inform risk awareness associated with minimally processed DLGVJs.

## 2 Material and methods

### 2.1 Preparation of conventional chard, collard green, and kale juices

Fresh conventional chard (*Beta vulgaris* subspecies *cycla*), collard greens (*Brassica oleracea* var. *viridis*), and kale (*Brassica oleracea* var. *acephala*) were purchased from a local supermarket in Davis, California. Multiple batches were obtained in the late summer and Fall 2024 to ensure representative sampling. Juice was prepared from the unwashed DLGVs using a commercial juicer (Breville^®^ Juice Fountain™ Plus, Australia) operated at high speed (13,000 rpm) for 5 min and there was no water added during juice extraction. The prepared dark leafy green vegetable juices (DLGVJs) were collected in sterile 50 mL Falcon™ tubes (VWR, Atlanta, GA) and stored at refrigerated temperature (4°C). Subsamples were collected and analyzed on days 0, 4, 7, 14, and 21 during cold storage (Guo et al., 2025a).

### 2.2 Physicochemical properties and microbiological changes in DLGVJs during cold storage

Color changes of DLGVJs including red-green (a*) and yellow-blue (b*) were determined by using a Hunter colorimeter (Hunter Colorflex EZ, United States), and the chroma (*C*) was calculated using the following Equation 1. The pH value of DLGVJs on each sampling day was tested by using a pH meter (FiveEasy Plus FP20, Mettler-Toledo, OH)


$$C\,\sqrt{a^{2}+b^{2}}$$ (1)

For the culture-dependent microbial analysis, samples were serially diluted in sterile phosphate-buffered saline (PBS; pH 7.4; Fisher Scientific, Hampton, NH, United States) and plated onto Plate Count Agar (PCA) and Dichloran Rose Bengal Chloramphenicol Agar (DRBC, BD Biosciences, Sparks, MD, United States), respectively, to obtain mesophilic total aerobic plate counts and total fungal counts. Two biological trials were conducted for each DLGVJ with three replicates taken at each sampling point during every trial, resulting in *n* = 6. PCA plates were incubated at 37°C for 24 h, while DRBC plates were incubated at room temperature (∼ 22°C) for 5 days before enumeration (Jeon et al., 2015).

### 2.3 DNA extraction and library preparation for microbiome analysis

Juice samples on Days 0 and 21 were used for DNA extraction. This aimed to profiling the original microbial populations present in three types of DLGVJs and in the spoiled DLGVJs. To extract the microbial DNA from DLGVJs, four 10 mL-subsamples were taken from each type of DLGVJs on the sampling day. Each subsample was centrifuged at 8,000 × *g* for 10 min at 4°C. The supernatant was discarded, and the resulting biomass was resuspended in 10 mL of 1 × PBS (Liao and Wang, 2021). The resulting cell pellet was washed twice with 1 × PBS, resuspended in 1 ml of 1 × PBS, and then transferred into a 2 mL microcentrifuge tube. The resuspended biomass was collected via centrifugation again and used for DNA extraction with the Qiagen PowerFood Microbial DNA Isolation Kit (QIAGEN, Valencia, CA) following the manufacturer’s instructions. DNA purity was assessed using a spectrophotometer (NanoDrop Technologies, DE) by measuring absorbance at 260 and 280 nm (Li et al., 2024). DNA concentration was quantified using a Qubit fluorometer (Thermo Fisher Scientific, MA). DNA quality was evaluated by loading 5μL of each sample on a 1.0% agarose gel and running at 110 V for 60 min (Ishido et al., 2010). A total of 48 high-quality DNA samples from fresh and spoiled DLGVJs were sent for sequencing. These samples comprised two independent biological replicates, each including four technical replicate juice subsamples per DLGVJ type (three DLGVJ types) at two sampling points (day 0 and 21). Technical replicates were processed independently through DNA extraction and sequencing. For downstream analysis, each subsample was retained as an individual data point to capture within-sample variability; however, these subsamples represent technical replicates derived from the same biological source. Accordingly, the analyses were primarily intended to describe overall microbial community patterns across juice types and storage conditions rather than to infer variability at the level of independent biological replicates. Amplicon libraries were prepared using the QIAseq 16S/ITS Panel Kit (QIAGEN), which targets the V3-V4 region of the bacterial 16S rRNA gene and the ITS region for fungal communities. The primers used for 16S rRNA are 5′-CCTACGGGNGGCWGCAG (forward) and 5′-GACTACHVGGGTATCTAATCC (reverse); primers targeting the ITS region are 5′-TCTTGGTCATTTAGAGGAAGTAA (forward) and 5′-GCTGCGTTCTTCATCGATGC (reverse) (available in the Qiagen Bioinformatics Manuals). The library size and concentration of 16S/ITS were assessed using a Bioanalyzer (BioTek, VT). Sequencing was performed on the Illumina MiSeq platform at the UC Davis Genome Center, generating 300-bp paired-end reads.

### 2.4 Bioinformatic analysis of 16S rRNA and ITS results

Raw sequences obtained from section 2.3 were quality-filtered using fastp (v0.19.6) (Chen et al., 2018) and subsequently merged with FLASH (v1.2.11) (Magoč and Salzberg, 2011). High-quality sequences were then denoised using the DADA2 plugin within the QIIME 2 pipeline (v2020.2) (Segata et al., 2011), applying the recommended parameters. Taxonomic classification was performed using the Naive Bayes consensus classifier in QIIME 2, with the SILVA database (for 16S rRNA) used for bacterial identification and UNITE 9.0 for fungal ITS sequences.

### 2.5 Isolation and Identification of lactic acid bacteria in spoiled DLGVJs

After 21 days of refrigerated storage, spoiled DLGVJs were serially diluted in sterile 1 × PBS. A 100 μL aliquot from each dilution was manually plated on de Man, Rogosa, and Sharpe (MRS) agar (BD Biosciences, Sparks, MD, United States). The plates were incubated anaerobically at 37 °C for 24–48 h to isolate lactic acid bacteria (LAB). To ensure broad representation of microbial diversity from each sample, each MRS agar plate was evenly divided into four equal sectors. From each sector, two colonies displaying distinct or representative morphologies were randomly selected using a sterile loop and transferred into 10 mL of MRS broth (Cotta et al., 2003; Dal Bello et al., 2003). The inoculated broths were incubated anaerobically at 37 °C for 24 h. Subsequently, each culture was streaked onto freshly prepared MRS agar plates to obtain isolated colonies and verify purity. Plates were incubated under identical anaerobic conditions for 24–48 h. A single well-isolated colony from each purified plate was then inoculated into 10 mL fresh MRS broth and incubated anaerobically at 37 °C for another 24 h to obtain active LAB cultures for downstream analyses (Endo et al., 2018). Genomic DNA from the isolated LAB strains was extracted using the Qiagen PowerFood Microbial DNA Kit (QIAGEN, Valencia, CA, United States) following the manufacturer’s instructions. The quality and concentration of the extracted DNA were evaluated as described in section 2.4. A total of 18 DNA samples were sequenced at the UC Davis Genome Center. Libraries were prepared with the Illumina Nextera DNA Flex Kit (Illumina Inc., San Diego, CA, United States) and sequenced as 150-bp paired-end reads on the Illumina HiSeq 4000 platform at the DNA Technologies and Expression Analysis Core. Quality of the LAB sequencing data was assessed using FastQC v0.11.8. Adapter trimming and quality filtering were performed with Trimmomatic v0.39 (Bolger et al., 2014). Genome assemblies were generated using SPAdes v3.15.4 (Bankevich et al., 2012), and assembly quality was evaluated with QUAST (Gurevich et al., 2013). Two isolates (LAB 110 and LAB 118) were excluded from further analysis due to poor assembly quality. Taxonomic identification of the assembled scaffolds was carried out using Kraken2 (Galaxy version 2.1.3 + galaxy1) (Wood and Salzberg, 2014). A phylogenetic tree based on Mash distances was generated using Mashtree (Katz et al., 2019), and tree visualization was performed with iTOL v5 (Letunic and Bork, 2021). *Leuconostoc mesenteroides* subsp. *mesenteroides* strain J18, isolated from fermented kimchi (GenBank accession: CP003101), and *Enterococcus faecium* strain YC07, isolated from fermented food jiangshui (BioProject: PRJNA1172820), were included as reference model strains for comparative genomic analysis and to anchor the phylogenetic tree.

### 2.6 Statistical analysis

Physicochemical properties (color and pH) and culture-dependent microbial counts, all experiments were conducted in duplicate with three replicates per trial (*n* = 6). Analysis of variance (ANOVA) followed by Duncan’s multiple range test was used to assess significant differences in microbial counts (APC and fungal counts) among DLGVJs prepared from different vegetables (chard, collard greens, and kale) and across different storage time points. R software (version 4.2.2; R Core Team and R Core Team, 2022) was used to perform the statistical analysis of culture-independent sequencing data. Alpha diversity was assessed using the Shannon index via the *phyloseq* package (McMurdie and Holmes, 2013). Beta diversity was evaluated using Principal Coordinate Analysis (PCoA) based on Bray-Curtis dissimilarities, implemented through the *vegan* package (version 2.6–4; Dixon, 2003). Statistical significance was determined using the Kruskal-Wallis test (Kruskal and Wallis, 1952) with Bonferroni correction (Chen et al., 2013). Pairwise comparisons were performed using the *pairwiseAdonis* package (version 0.4) with Bonferroni correction (Martinez Arbizu, 2020).

## 3 Results

### 3.1 Changes of physicochemical properties in conventional DLGVJs during 21-day refrigerated storage

**FIGURE 1:**
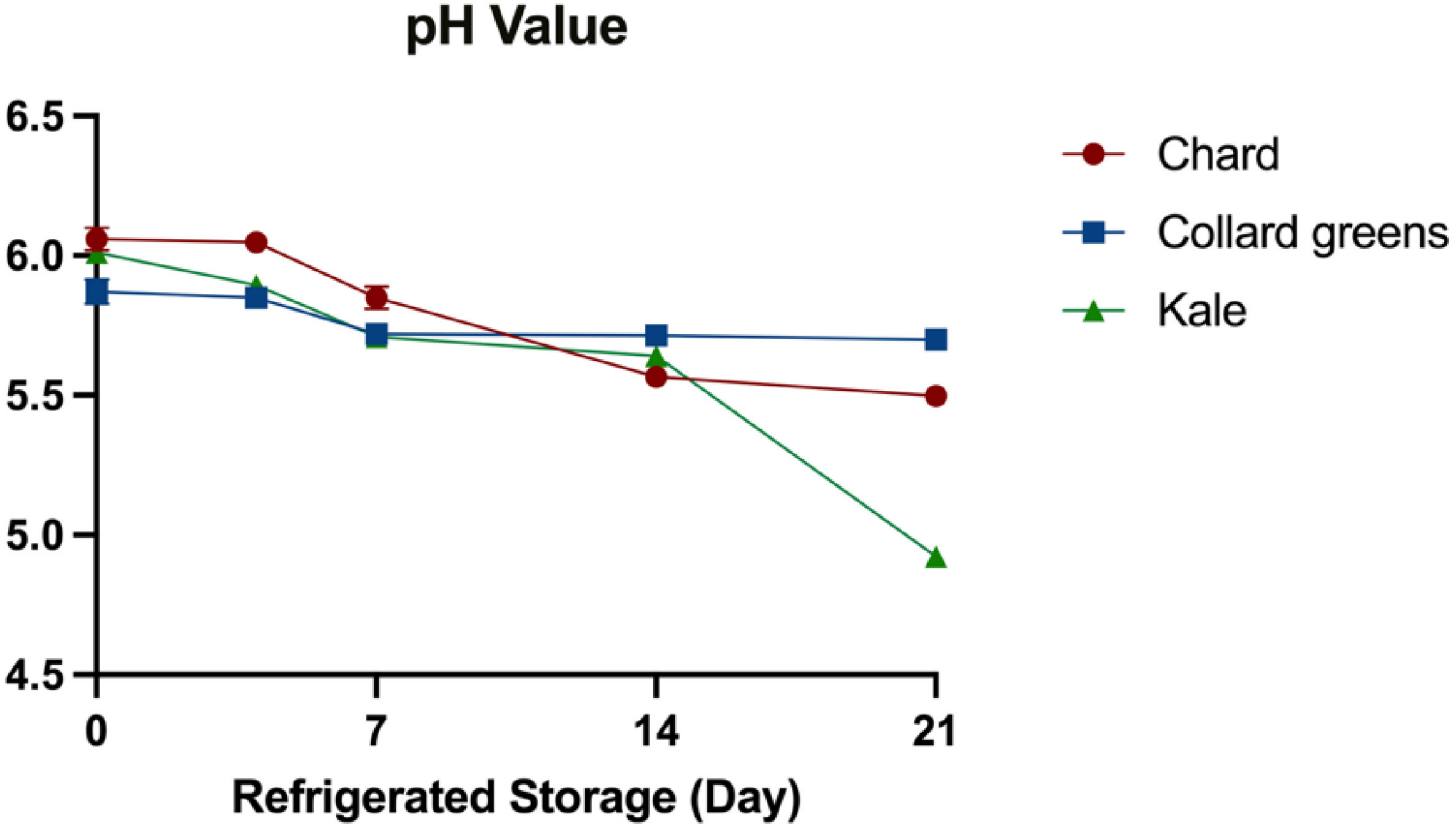
pH changes of conventional DGLVJs during 21-day refrigerated storage.

Figure 1 illustrates the pH changes in three conventional DLGVJs during 21 days of refrigerated storage. On day 0, the initial pH values of fresh chard, collard greens, and kale juices were 6.06 ± 0.04, 5.87 ± 0.04, and 6.01 ± 0.03, respectively. After 4 days of refrigeration, a significant decrease (*P* < 0.05) in pH was observed only in kale juice (5.89 ± 0.02), while the pH values of chard and collard greens remained relatively stable. However, by day 7, both chard and collard greens juices exhibited noticeable pH declines, reaching 5.85 ± 0.04 and 5.72 ± 0.03, respectively. A continuous decreasing trend in pH was observed for chard and kale juices from day 7 to day 21. The most significant pH reduction occurred in kale juice, which dropped from 6.01 ± 0.03 to 4.92 ± 0.02, while collard greens juice showed limited change, declining only slightly from 5.87 ± 0.04 to 5.70 ± 0.02.

The changes in color profile (a* and C*) of conventional DLGVJs during refrigerated storage are summarized in Table 1. On day 0, chard, collard greens, and kale juices exhibited negative a* values (−4.77 ± 0.04, –12.10 ± 0.23, and –12.05 ± 0.21, respectively), consistent with their characteristic green color. After 21 days of refrigerated storage, a significant increase (*P* < 0.05) in a* was observed in kale juice, reaching −11.30 ± 0.16, indicating a shift toward a less intense greenness. In contrast, no noticeable changes in a* were observed for chard or collard greens juices (−5.15 ± 0.24 and −11.30 ± 0.16, respectively), suggesting relatively stable green color during storage. The initial C* values differed among juices, with values of 11.74 ± 0.35 for chard, 23.95 ± 0.42 for collard greens, and 25.72 ± 0.38 for kale juice, reflecting differences in initial color saturation. Overall, an increasing trend in C* values was observed in all three DLGVJs over the 21-day refrigerated storage, indicating progressively darker and more saturated green appearance. Significant increases (*P* < 0.05) in C* values were detected in chard and collard greens juices as early as day 4, reaching 16.37 ± 0.22 and 24.74 ± 0.34, respectively. In kale juice, although no significant differences were observed during the first 7 days, C* values gradually increased to 26.27 ± 0.20 by day 21. By the end of storage, C* values increased to 20.66 ± 0.13 for chard juice and 26.13 ± 0.26 for collard greens juice.

**TABLE 1:** Color changes^1^ of conventional DLGVJs during 21-day storage at refrigerated temperature.

Color parameters	DLGVJs	Storage time/day				
		0	4	7	14	21
a*	Chard	−4.77 ± 0.04^ab^	−4.76 ± 0.13^ab^	−4.61 ± 0.49^a^	−5.18 ± 0.16^b^	−5.15 ± 0.24^ab^
	Collard greens	−12.10 ± 0.23^a^	−11.91 ± 0.11^a^	−11.84 ± 0.29^a^	−11.89 ± 0.20^a^	−11.70 ± 0.26^a^
	Kale	−12.05 ± 0.21^a^	−12.02 ± 0.16^a^	−11.82 ± 0.29^a^	−11.81 ± 0.13^a^	−11.30 ± 0.16^b^
C*	Chard	11.74 ± 0.35^a^	16.37 ± 0.22^b^	17.00 ± 0.74^b^	19.95 ± 0.29^c^	20.66 ± 0.18^c^
	Collard greens	23.95 ± 0.42^a^	24.74 ± 0.34^b^	23.99 ± 0.50^a^	25.11 ± 0.43^b^	26.13 ± 0.26^c^
	Kale	25.72 ± 0.38^ab^	26.15 ± 0.42^bc^	25.31 ± 0.44^a^	26.64 ± 0.30^c^	26.27 ± 0.20^bc^

^1^For each color change, means within each group time with different lowercase letters are significantly different during storage t (*P* < 0.05).

### 3.2 Culture-dependent changes in conventional DLGVJs during 21-day refrigerated storage

**FIGURE 2:**
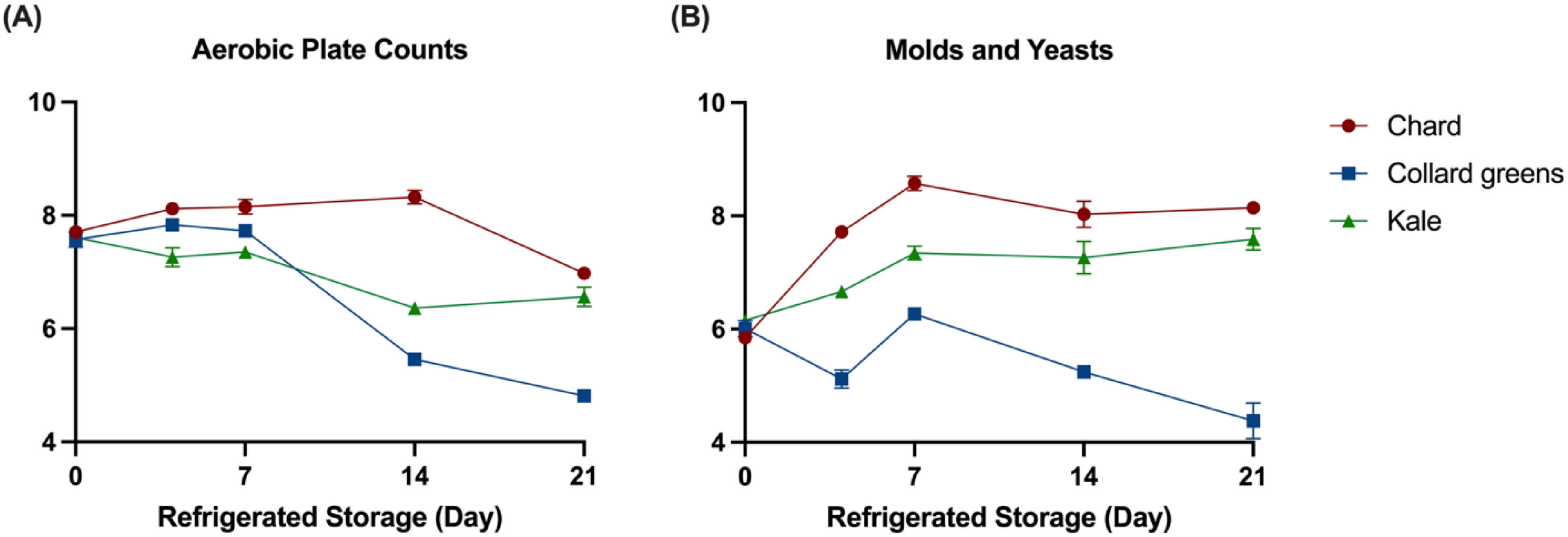
Changes of total aerobic bacterial **(A)** and total fungal populations **(B)** in conventional DLGVJs stored at refrigeration for 21-day.

The changes in total aerobic plate counts (APC) and fungal populations (molds and yeasts) during storage are presented in Figure 2. The initial APC levels of chard, collard greens, and kale juices were 7.71 ± 0.03, 7.57 ± 0.14, and 7.61 ± 0.09 Log CFU/mL, respectively (Figure 2A). By day 4, a notable increase in APC (*P* < 0.05) was observed in both chard and collard greens juices, reaching 8.12 ± 0.08 and 7.83 ± 0.05 Log CFU/mL, respectively. In chard juice, APC continued to rise, peaking at 8.32 ± 0.12 Log CFU/mL on day 14, followed by a significant decline to 6.98 ± 0.03 Log CFU/mL on day 21. In collard greens juice, APC remained relatively stable between day 4 and day 7, then decreased markedly to 4.81 ± 0.10 Log CFU/mL by day 21. In contrast, a continuous decreasing trend in APC was observed in kale juice, with levels decreasing steadily to 6.56 ± 0.17 Log CFU/mL by the end of the storage period.

Fungal population changes in conventional DLGVJs during refrigerated storage are shown in Figure 2B. The initial fungal populations in chard, collard greens, and kale juices were 5.85 ± 0.03, 6.01 ± 0.15, and 6.15 ± 0.03 Log CFU/mL, respectively. An increasing trend was observed in chard and kale during the first 7 days of storage, reaching 8.57 ± 0.13 and 7.35 ± 0.12 Log CFU/mL, respectively. For collard green juice, after a short decline observed on day 4, the total fungal counts remained at 6.27 ± 0.02 level on day 7, with no significant difference. From day 7 to day 21, a ∼ 2 Log reduction was observed in collard green juice while the levels of fungal counts didn’t change quite much in chard and kale juices.

### 3.3 Bacterial and fungal communities present in conventional DLGVJs

**FIGURE 3:**
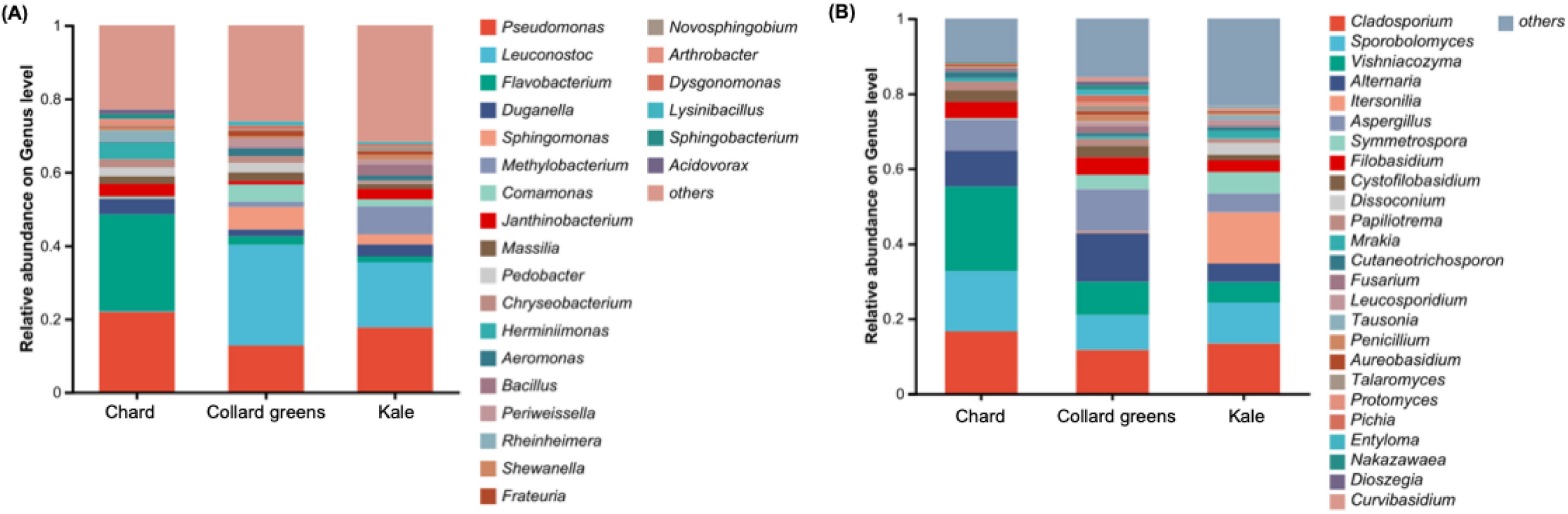
Bacterial and fungal communities in conventional Chard, Collard Greens, and Kale juices on Day 0. **(A)** Relative abundances of bacterial **(A)** and fungal **(B)** genera. Genera with relative abundances below 0.01% are grouped as “Other.”

Microbial communities present in fresh DLGVJs on day 0 are presented in Figure 3, and top five dominant bacterial and fungal genera in fresh DLGVJs with relative abundance is shown in Table 2. A total of 24 bacterial and 25 fungal genera were identified from three juices with their relative abundance over 0.01% (Figures 3A,B). In fresh chard juice, the dominant bacterial genera identified were *Flavobacterium* (26.45%), *Pseudomonas* (21.89%), *Herminiimonas* (4.38%), *Duganella* (3.41%), and *Janthinobacterium* (3.40%). In fresh collard greens juice, the most abundant bacterial genera were *Leuconostoc* (27.45%), *Pseudomonas* (12.77%), *Sphingomonas* (6.14%), *Comamonas* (4.79%), and *Pedobacter* (2.62%). For kale juice, *Leuconostoc* (17.83%), *Pseudomonas* (17.69%), *Methylobacterium* (7.61%), *Duganella* (3.19%), and *Bacillus* (3.10%) were identified as the predominant bacterial genera. Regarding the fungal communities, the dominant genera in chard juice were *Vishniacozyma* (22.21%), *Cladosporium* (16.48%), *Sporobolomyces* (16.38%), *Alternaria* (9.36%), and *Aspergillus* (8.42%). In fresh collard greens juice, *Alternaria* (13.28%), *Cladosporium* (12.42%), *Aspergillus* (11.28%), *Sporobolomyces* (8.86%), and *Vishniacozyma* (8.35%) were the top five genera. For fresh kale juice, *Itersonilia* (14.37%), *Cladosporium* (12.99%), *Sporobolomyces* (10.71%), *Vishniacozyma* (5.92%), and *Aspergillus* (5.82%) were identified as the dominant fungal communities (Table 2).

**TABLE 2:** Top five dominant bacterial and fungal genera present in Day 0 conventional DLGVJs.

DLGVJs	Top five genera and their relative abundance identified from DLGVJs					
Chard	Bacterial	*Flavobacterium*	*Pseudomonas*	*Herminiimonas*	*Duganella*	*Janthinobacterium*
		26.45%	21.89%	4.38%	3.41%	3.41%
	Fungal	*Vishniacozyma*	*Cladosporium*	*Sporobolomyces*	*Altheria*	*Aspergillus*
		22.21%	16.48%	16.38%	9.36%	8.42%
Collard greens	Bacterial	*Leuconostoc*	*Pseudomonas*	*Sphingomonas*	*Comamonas*	*Pedobacter*
		27.45%	12.77%	6.14%	4.79%	2.62%
	Fungal	*Alternaria*	*Cladosporium*	*Aspergillus*	*Sporobolomyces*	*Vishniacozyma*
		13.28%	12.42%	11.28%	8.86%	8.35%
Kale	Bacterial	*Leuconostoc*	*Pseudomonas*	*Methylobacterium*	*Duganella*	*Bacillus*
		17.83%	17.68%	7.61%	3.19%	3.10%
	Fungal	*Itersonilia*	*Cladosporium*	*Sporobolomyces*	*Vishniacozyma*	*Aspergillus*
		14.37%	12.99%	10.71%	5.92%	5.82%

**FIGURE 4:**
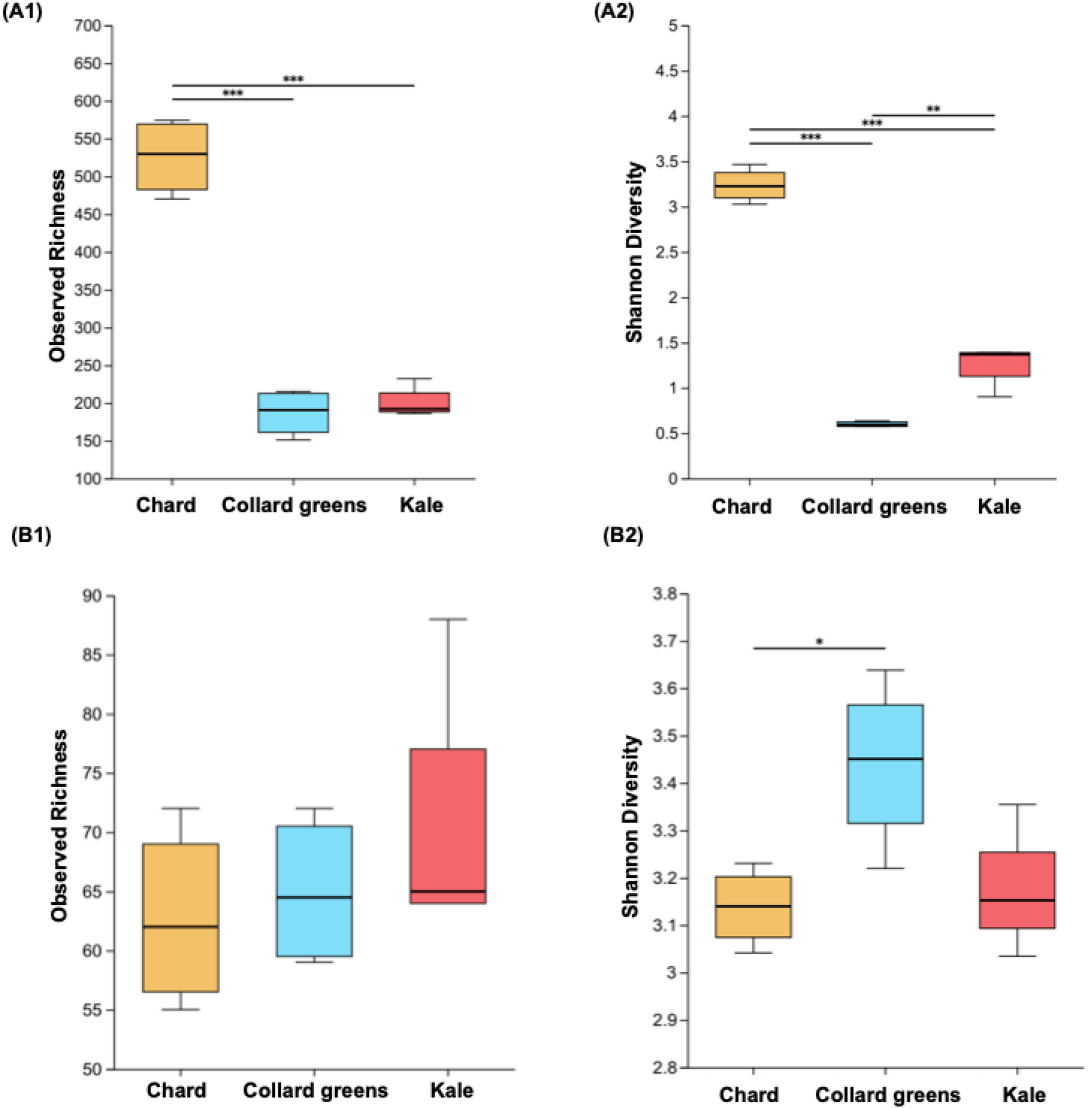
Observed and Shannon indexes of bacterial **(A1,A2)** and fungal **(B1,B2)** communities present in conventional chard, collard green, and kale juices (*n* = 8) on Day 0. Notes: ***p < 0.001, **p < 0.01, *p < 0.05.

**FIGURE 5:**
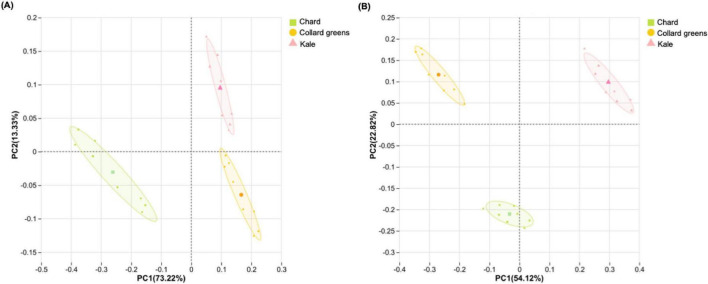
Principal coordinates analysis (PCoA) of the bacterial **(A)** and fungal **(B)** communities present in conventional DLGVJs on Day 0. Each point represents a subsample (2 biological replicates, each biological replicate conducting with 4 technicals; *n* = 8 per group). The centroid of each ellipse represents the group mean; the shaded polygons, indicating the 95% confidence region of each cluster, are applied to differentiate sample types.

Figure 4 presents the alpha diversity analysis of three conventional DLGVJs on day 0. Bacterial richness, quantified as observed richness (number of distinct taxa), showed significant differences between chard and collard greens, and between chard and kale (*p* < 0.001; Figure 4A1). Regarding Shannon diversity, all pairwise comparisons among the three juices showed significant differences (*p* < 0.01; Figure 4A2). For fungal communities in DLGVJs, no significant differences were observed in richness among the three juices (Figure 4B1). However, a significant difference in Shannon diversity was found between chard and collard greens (*p* < 0.05; Figure 4B2). Principal coordinate analysis (PCoA) based on Bray-Curtis dissimilarity demonstrated distinct clustering of chard, collard greens, and kale juices on day 0 (Figure 5), indicating differences in overall microbial community composition among juice types. Pairwise comparisons of bacterial communities revealed significant differences between chard and collard greens (*p* = 0.023), chard and kale (*p* = 0.027), and collard greens and kale (*p* = 0.031). Similarly, fungal community compositions differed significantly among the three juices, with *p*-values of 0.026, 0.031, and 0.030 for chard versus collard greens, chard versus kale, and collard greens versus kale, respectively (Supplementary Table S1). These analyses reflect differences among juice types at the community level rather than variability among individual replicate samples.

### 3.4 Changes of microbial communities in conventional DLGVJs after 21 days of refrigerated storage

**FIGURE 6:**
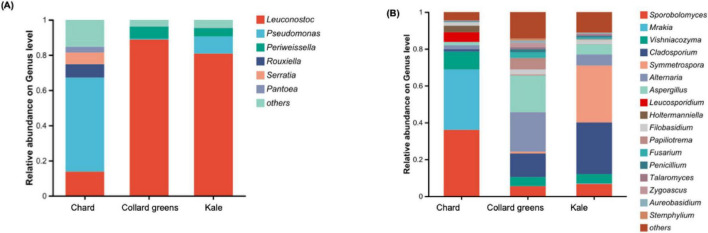
Bacterial and fungal communities in conventional Chard, Collard Greens, and Kale juices on Day 21. **(A)** Relative abundances of bacterial **(A)** and fungal **(B)** genera. Genera with relative abundances below 0.01% are grouped as “Other.”

After 21-day refrigerated storage, the microbial communities in spoiled DLGVJs are shown in Figure 6, the numbers of the dominant bacterial and fungal genera (with relative abundance > 0.01%) decreased to 6 and 17, respectively. By Day 21, noticeable phase separation had occurred in the DLGVJs, with a clear liquid layer forming at the top and biomass settling at the bottom. A distinct white microbial mat was observed at the interface between the two layers. Additionally, the juices exhibited evident spoilage characteristics, including sour or off-odors and visible color deterioration. In spoiled chard juice, *Pseudomonas* became the dominant bacterial genus, with its relative abundance increased from 21.89 to 53.37%. Other major genera in spoiled chard juice included *Leuconostoc* (13.75%), *Rouxiella* (7.65%), *Serratia* (6.62%), and *Massilia* (3.29%). In spoiled collard greens juice, *Leuconostoc* showed a dramatic increase to 88.73%, as the most dominant genus and *Periweissella* also grew significantly from below 0.01 to 6.91%, other dominant bacterial communities were *Pseudomonas*, *Comamonas*, *Aeromonas* with the relative abundance was 0.50, 0.25, and 0.19% respectively. In spoiled kale juice, *Leuconostoc* and *Pseudomonas* remained dominant, but their relative abundances shifted notably: *Leuconostoc* increased from 17.83 to 80.84%, while *Pseudomonas* declined to 9.65%. Other dominant genera included *Periweissella* (4.84%), *Bacillus* (0.62%), and *Comamonas* (0.31%). Overall, a clear shift from more diverse microbial communities at Day 0 to dominance by a limited number of taxa at Day 21 was observed across all DLGVJs. This transition was characterized by increased relative abundance of *Leuconostoc* in collard greens and kale juices and *Pseudomonas* in chard juice, along with changes in dominant genera across juice types.

Dominant fungal communities in DLGVJs on day 21 are shown in Table 3. In spoiled chard juice, *Sporobolomyces* increased markedly from 16.38 to 37.68%, and *Mrakia* grew significantly from below 0.01 to 31.88%. *Vishniacozyma*, initially the most abundant genus in fresh chard juice, declined sharply to 9.00%. *Leucosporidium* (5.44%) and *Holtermanniella* (3.23%) also emerged among the top five fungal genera. In spoiled collard greens juice, *Alternaria* remained the dominant fungal genus, with its relative abundance increasing from 13.28 to 20.88%. A notable rise was also observed in *Aspergillus*, which grew from 11.28 to 19.71%. Compared to day 0, *Cladosporium* showed minimal change (from 12.42 to 12.73%), *Papiliotrema* (6.93%) and *Sporobolomyces* (5.34%) were the dominant fungal genera. *Symmetrospora* with the highest relative abundance (30.17%) was found in spoiled kale juice, despite being absent in fresh kale juice. *Cladosporium*, initially the most abundant in fresh kale juice, increased from 12.99 to 22.64%. *Aspergillus* also showed a rising trend, reaching 6.59%. In contrast, *Sporobolomyces* declined from 10.71 to 7.38%. *Alternaria* (7.85%) became a dominant genus in spoiled kale juice, replacing *Vishniacozyma*.

**TABLE 3:** Top five dominant bacterial and fungal genera present in Day 21 conventional DLGVJs.

DLGVJs	Top five genera and their relative abundance identified from DLGVJs					
Chard	Bacterial	*Pseudomonas*	*Leuconostoc*	*Rouxiella*	*Serratia*	*Massilia*
		53.37%	13.75%	7.65%	6.62%	3.29%
	Fungal	*Sporobolomyces*	*Mrakia*	*Vishniacozyma*	*Leucosporidium*	*Holtermanniella*
		37.68%	31.88%	9.00%	5.44%	3.23%
Collard greens	Bacterial	*Leuconostoc*	*Periweissella*	*Pseudomonas*	*Comamonas*	*Aeromonas*
		88.73%	6.91%	0.50%	0.25%	0.19%
	Fungal	*Alternaria*	*Aspergillus*	*Cladosporium*	*Papiliotrema*	*Sporobolomyces*
		20.88%	19.71%	12.73%	6.93%	5.34%
Kale	Bacterial	*Leuconostoc*	*Pseudomonas*	*Periweissella*	*Bacillus*	*Comamonas*
		80.84%	9.65%	4.84%	0.62%	0.31%
	Fungal	*Symmetrospora*	*Cladosporium*	*Alternaria*	*Sporobolomyces*	*Aspergillus*
		30.17%	22.64%	7.85%	7.38%	6.59%

**FIGURE 7:**
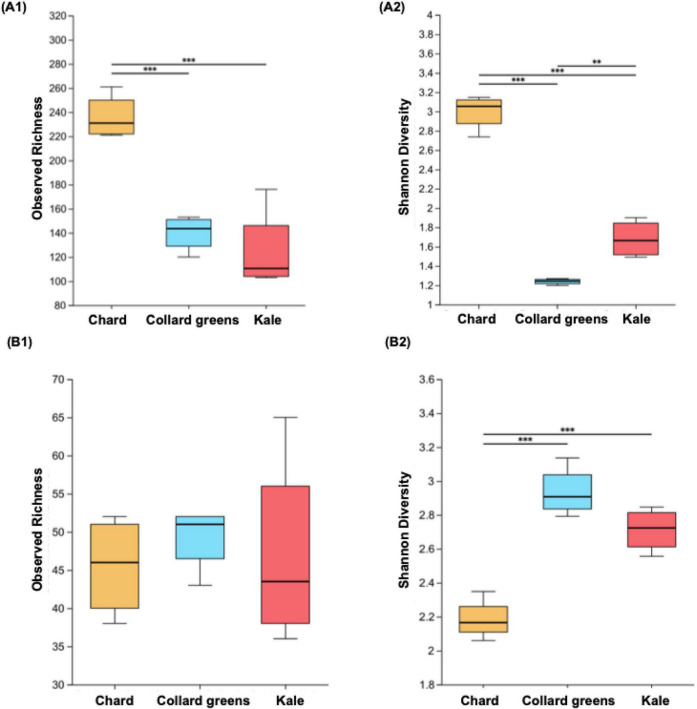
Observed and Shannon indexes of bacterial **(A1,A2)** and fungal (**B1,B2)** communities present in conventional chard, collard green, and kale juices (*n* = 8) on Day 21. Notes: ***p < 0.001, **p < 0.01.

**FIGURE 8:**
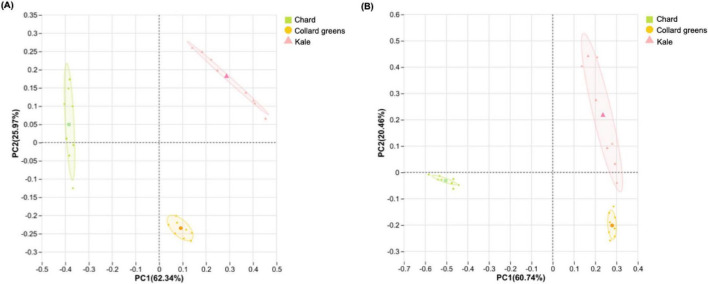
Principal coordinates analysis (PCoA) of the bacterial **(A)** and fungal **(B)** communities present in conventional DLGVJs on Day 21. Each point represents a subsample (2 biological replicates, each biological replicate conducting with 4 technicals; *n* = 8 per group). The centroid of each ellipse represents the group mean; the shaded polygons, indicating the 95% confidence region of each cluster, are applied to differentiate sample types.

Figure 7 presents the alpha diversity of bacterial and fungal communities in spoiled DLGVJs. Significant differences in bacterial richness were observed between chard and collard greens, and between chard and kale (*p* < 0.001), while no difference was found between collard green and kale juices (Figure 7A1). Shannon diversity differed significantly among all juice groups (*p* < 0.01; Figure 7A2). For fungal communities, no significant differences in richness were observed among three juices (Figure 7B1), but Shannon diversity differed significantly between chard and collard greens, as well as between chard and kale (*p* < 0.001; Figure 7B2). Principal Coordinates Analysis (PCoA) of bacterial and fungal communities in spoiled conventional DLGVJs on day 21 is shown in Figure 8. Distinct clustering patterns were observed among the three juices was found (Figure 8). Pairwise comparisons of bacterial communities revealed significant differences between chard and collard greens (*p* = 0.022), and chard and kale (*p* = 0.028), but not between collard greens and kale (*p* = 0.91). For fungal communities, significant differences were found among all pairwise comparisons: chard vs. collard greens (*p* = 0.033), chard vs. kale (*p* = 0.030), and collard greens vs. kale (*p* = 0.031) (Supplementary Table S2).

### 3.5 Identification of lactic acid bacteria isolated from spoiled DLGVJs

**FIGURE 9:**
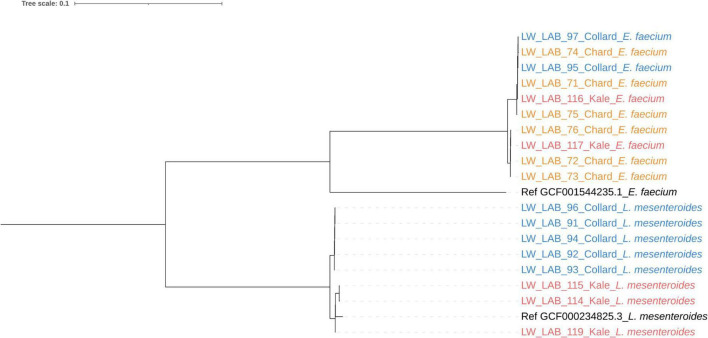
Whole-genome-based phylogenetic tree of lactic acid bacteria (LAB) isolated from spoiled conventional DLGVJs and model strains (*E. faecium* YC 07 and *L. mesenteroides* J18), constructed using the Mashtree algorithm based on pairwise Mash distances. Distinct clades represent major LAB genera, including *Leuconostoc* and *Enterococcus*. The tree demonstrates high genomic similarity within species clusters and clear divergence among genera, reflecting the taxonomic and ecological diversity of LAB associated with juice spoilage.

Table 4 summarizes the lactic acid bacteria (LAB) isolated from spoiled DLGVJs. Among the total 18 isolates obtained, two major LAB species were identified: *Enterococcus faecium and Leuconostoc mesenteroides*. Among them, *E. faecium* was the most prevalent, with six, two, and two strains isolated from spoiled chard, collard greens, and kale juices, respectively. *L. mesenteroides* was detected in collard greens and kale juices, with five and three isolates. As illustrated in Figure 9, whole-genome phylogenetic analysis using MashTree further confirmed the species-level assignments. Two clearly separated clades were observed: one comprising *E. faecium* isolates that clustered tightly with the food-associated reference strain YC07, and the other containing *L. mesenteroides* isolates positioned near the reference strain J18. The *E. faecium* isolates clustered tightly with the food-associated reference strain YC07, indicating a high degree of genomic similarity among isolates recovered from food made with related materials, in this case vegetables (Cao et al., 2025). In contrast, *L. mesenteroides* isolates formed a distinct clade positioned near the reference strain J18 but exhibited slightly greater genomic dispersion, which may reflect adaptation to differing physicochemical conditions in collard greens and kale juices during storage.

**TABLE 4:** Lactic acid bacteria isolated from spoiled conventional DLGVJs at species level.

DLGVJs	*Enterococcus faecium*	*Leuconostoc mesenteroides*
Chard	6	0
Collard greens	2	5
Kale	2	3

## 4 Discussion

In recent years, the market for dark leafy green vegetable juices (DLGVJs) has grown rapidly due to their rich nutritional profile and health benefits (Kumar et al., 2020; Satheesh et al., 2020). However, their darker color and neutral pH make them more susceptible to microbial spoilage (Salehi, 2021). As a novel food matrix, research on dark leafy green vegetables (DLGVs) and DLGVJs remains limited. Over the past 2 years, the research team has investigated the microbial safety of DLGVJs, including the microbial composition in organic DLGVJs during refrigerated storage and the fate of foodborne pathogens in DLGVJs stored at various temperatures (Guo et al., 2025a,b). Conventional DLGVs represent the larger segment of the market compared to organic production, the microbial safety of DLGVJs made with conventional DLGVs remains uninvestigated. Moreover, previous studies have indicated distinct microbial differences between organic and conventional produce (Jensen et al., 2013; Leff and Fierer, 2013), underscoring the need to characterize microbial profile in both types of DLGVJs during refrigerated storage. A comprehensive microbial profile of conventional DLGVJs will provide critical scientific evidence to improve their quality and safety.

pH, as a key indicator of juice quality and stability, declined during 21-day refrigerated storage (Figure 1). The initial pH value of conventional ranged from 5.87 ± 0.04 to 6.06 ± 0.04; while in our previous study on organic DLGVJs, a similar pH range (5.87–6.08) was observed. By the end of 21-day refrigerated storage, the pH in spoiled conventional juice, ranging from 4.92 ± 0.02 to 5.70 ± 0.02, lower than the pH of the spoiled organic juice (Guo et al., 2025a). This difference between organic and conventional juices might be caused by the different buffering capacity. Although there has not been any study directly analyzing the buffer capacity of organic and conventional DLGVs, it has been shown that the buffering capacity of food correlated with the protein content as well as the content of aspartic and glutamic acid (Al-Dabbas et al., 2010). DLGVs, such as kale, is rich in aspartic and glutamic acid (Lisiewska et al., 2008). The pH of conventional collard greens juice remained stable from day 7 to day 21. This pH stability of collard greens juice across various storage temperatures (4, 10, and 23°C) was also reported by Guo et al. (2025b). The higher concentration of minerals (e.g. calcium, magnesium, and potassium) in collard greens by comparison to other two DLGVs (FoodData, USDA, nd), which may contribute to stronger buffer capacity, resisting changes in pH when stored at refrigerates temperatures (Friedman and Jürgens, 2000).

Color is a key determinant of consumer preference for leafy greens, and previous studies indicate that a greener appearance is positively correlated with consumers’ perceptions of freshness and overall quality (Dinnella et al., 2014; Swegarden et al., 2019). In this study, the a* value (red-green axis) increased during storage, accompanied by higher chroma (C), indicating reduced greenness and progressive color saturation in conventional DLGVJs (Table 1); a similar trend was observed in organic DLGVJs. The observed changes in color profiles during storage may be attributed to the degradation of pigments (e.g., chlorophyll) and enzymatic browning. Chlorophyll, which imparts the characteristic green color to DLGVJs, can degrade into colorless compounds such as pheophorbide, contributing to the increase in a* values (Heaton and Marangoni, 1996). Additionally, enzymatic browning driven by polyphenol oxidase (PPO) and peroxidase (POD) activity may result in color dullness, as reflected by the increase in chroma (C) values during refrigerated storage (Singh et al., 2018). In this study, the greatest increase of chroma was observed in chard juice during refrigerated storage. Swiss chard, especially the red or rainbow varieties, contains betalains (betacyanins and betaxanthins) in addition to chlorophyll, and degradation or transformation of these pigments may lead to changes in chroma over time (Azeredo, 2009; Gaur et al., 2006).

According to the culture-dependent analysis, the initial aerobic plate counts (APC) in conventional DLGVJs were higher than those in the organic samples, while the initial fungal populations were similar between the two groups (Guo et al., 2025a). During 21-day refrigerated storage, APC in all three conventional DLGVJs showed an overall decreasing trend, whereas fungal populations initially increased and subsequently declined (Figure 2). In contrast, APC in organic DLGVJs increased during the early storage period before declining, while fungal populations decreased initially and then increased over time (Guo et al., 2025a). The difference in the fate of native microbes may be attributed by various factors. First, the distinct concentrations of antimicrobial components between organic and conventional DLGVs. Phenolic acids, recognized as key antimicrobial components in DLGVs (Ayaz et al., 2008), can vary in concentration depending on the cropping system. Zhao et al. (2009) reported that organic farming produced significantly higher phenolic concentrations in pac choi (*Brassica rapa* L. *chinensis*) compared to conventional fertilization, under both high tunnel and open field conditions. For instance, *Pseudomonas*, the dominant bacterial genus presents in both organic and conventional DLGVJs can be effectively inhibited by phenolic acids such as benzoic, phenylacetic, and phenylpropionic acids extracted from vegetables (Cueva et al., 2010; Yun et al., 2019). Secondly, different microorganisms associated specifically with fresh produce cultivated under conventional or organic sources may react differently to various stresses during refrigerated storage Jensen et al. (2013) and Leff and Fierer (2013). For instance, Rossetti et al. (2023) identified only *Saccharomyces* spp. *from* conventionally grown grapes. Data from Aguilera et al. (2007) and (Garcia-Rios et al., 2016) demonstrated that *Saccharomyces* could survive for long period of time under low-temperature conditions (<10°C). The findings based on culture-dependent approaches also highlight the importance of identifying microbial taxa in conventional DLGVJs by using culture-interdepend approaches as many microorganisms cannot be cultured/checked with traditional methods.

16S rRNA and ITS sequencing were used to investigate changes in microbial composition in conventional DLGVJs (Figures 3–8). In fresh conventional DLGVJs, *Pseudomonas* was identified as the dominant bacterial genus shared among all three juice types (Table 2), which has been demonstrated responsible for the leafy green spoilage (Raposo et al., 2016). While *Leuconostoc* was the most abundant bacterial genus in fresh conventional collard greens and kale juices, it was not detected as a dominant genus in chard juice. The fungal communities in fresh conventional DLGVJs showed high similarity among the three juice types, with only minor distinctions. For example, *Itersonilia* was uniquely identified in fresh kale juice, where it had the highest relative abundance. Notably, *Itersonilia perplexans* Derx 1948 is a known phytopathogen that affects various crops including lettuce and tulip by infecting leafy tissues, disrupting physiological processes, and triggering symptoms such as brown spots and wilting which can reduce yield and quality (Boekhout, 1991; Pilkington et al., 2023). When compared to the microbial communities in fresh organic DLGVJs, *Herminiimonas*, *Pedobacter*, and *Duganella* were dominant bacterial genera uniquely identified in conventional DLGVJs. The fungal compositions were largely shared between the two groups, except for *Itersonilia*, which was only found in conventional samples. These differences in microbial profiles between organic and conventional fresh DLGVJs may lead to distinct spoilage pathways during refrigerated storage. For example, *Pedobacter*, detected primarily in conventional juices, has been recognized as a predominant spoilage genus in fresh white button mushrooms (*Agaricus bisporus*) (Qiu et al., 2019; Yang et al., 2019) and identified as spoilage-associated in box-packaged sturgeon fillets (Zhang et al., 2020). Its metabolic activity can generate off-odors through the conversion of volatile compounds, potentially contributing to the distinct odor characteristics observed in the two spoiled juice types.

After 21-day refrigerated storage, *Leuconostoc* emerged as the dominant spoilage bacterial genus in DLGVJs based on the sequencing results (Figure 6), with relative abundances exceeding 80% in collard greens and kale juices (Table 3), which also found in spoiled organic DLGVJs but with less relative abundance (Guo et al., 2025a). Paillart et al. (2017) reported that the rapid growth of *Leuconostoc* species was associated with the development of a sour off-odor, characterized by the accumulation of acetic and lactic acids, in modified-atmosphere packaged lettuce stored at 7°C. Similarly, Ioannidis et al. (2018) demonstrated that spoilage under anaerobic conditions in modified-atmosphere packaged iceberg lettuce was dominated by *Leuconostoc* spp. Consistent with these findings, visible gas formation accompanied by sour off-odors was observed in conventional DLGVJs toward the end of refrigerated storage in the present study, although gas production was not quantitatively measured. Together, these observations support the role of certain *Leuconostoc* species as important spoilage-associated microorganisms across diverse vegetable-based food matrices.

Interestingly, in this study, the relative abundance of *Leuconostoc* was higher in spoiled collard greens and kale juices when compared among three DLGVJs. This difference may be attributed to the distinct phenolic profiles associated with their taxonomic families; chard belongs to the *Amaranthaceae* family, while kale and collard greens are members of the *Brassicaceae* family. Lin and Harnly (2009) demonstrated that kale and collard greens share highly similar phenolic profiles, suggesting that the fate of microorganisms in these two DLGVJs may also be similar during storage. In addition, kale and collard greens contain higher levels of kaempferol, a flavonoid with reported antimicrobial activity, while only trace amounts are present in chard (Gamba et al., 2021; Huang et al., 2007). Moreover, the inhibitory effects of certain *Leuconostoc* species against foodborne pathogens in leafy greens have been reported by Trias et al. (2008a) and Trias et al. (2008b), as evidenced by their ability to suppress the growth of foodborne pathogens in collard greens and kale juices under various storage temperatures (Guo et al., 2025b), while indicated the growth trend in chard juice when stored at the same temperature.

Whole-genome phylogenetic analysis provided deeper insight into the diversity of lactic acid bacteria (LAB) associated with DLGVJ spoilage. Two well-defined clusters corresponding to *Enterococcus faecium* and *Leuconostoc mesenteroides* were observed, supporting the species-level identifications obtained through sequencing (Figure 9 and Table 4). *E. faecium* isolates recovered from spoiled DLGVJs exhibited close genomic similarity to the food-associated model strain *E. faecium* YC07, originally isolated from fermented Jiangshui. According to Cao et al. (2025), strain YC07 is capable of biodegrading inosine and guanosine into smaller metabolites such as hypoxanthine and xanthine. Notably, hypoxanthine has been recognized as a spoilage-associated biomarker in multiple food matrices (Liu et al., 2025; Wang et al., 2025). The strong genomic relatedness observed suggests that *E. faecium* strains from spoiled DLGVJs may share a similar spoilage niche and metabolic potential during refrigerated storage. In contrast, *L. mesenteroides* isolates exhibited greater genomic diversity, indicating possible adaptation to distinct juice environments. Compared with *L. mesenteroides* J18 isolated from fermented kimchi (Jung et al., 2012), isolates from spoiled kale juice showed higher genomic similarity. As an obligate heterofermentative LAB, *L. mesenteroides* generates organic acids and other metabolites by fermenting sugars. Interestingly, Hye Baek et al. (2024) reported that the fermentation end-products of *L. mesenteroides* J18 vary depending on the available carbon sources. This indicates that differences in sugar composition among DLGVJs may influence spoilage-associated metabolic pathways and the resulting profiles of fermentation end-products.

Such metabolite-driven differences may further shape the microbial ecology during storage. The accumulation of organic acids and other fermentation products can create a selective microenvironment that favors acid-tolerant microorganisms such as LAB, while inhibiting less tolerant taxa (Yang et al., 2021). Under these stress conditions, some microorganisms detected by amplicon sequencing may persist in a viable but non-culturable (VBNC) or metabolically inactive state, limiting their recovery through standard cultivation methods. In addition, the use of MRS agar under anaerobic conditions further selects for LAB, and the rapid growth and acid production of these organisms during isolation may suppress competing taxa (Dong et al., 2020; Pazos-Rojas et al., 2023). Together, these findings suggest that metabolite accumulation during spoilage not only drives microbial community succession in DLGVJs but also contributes to the observed discrepancies between sequencing-based and culture-based approaches. These results provide a genomic basis for understanding the metabolic roles of LAB in DLGVJ spoilage. Future studies integrating comparative genomics with functional validation will be essential to identify specific genetic determinants underlying spoilage-associated metabolism and microbial adaptation in DLGVJs.

The spoiled conventional DLGVJs harbored similar core fungal communities. *Sporobolomyces* remained the predominant fungal genus in both fresh and spoiled conventional DLGVJs (Table 4), as well as in their organic counterparts (Guo et al., 2025a). However, certain fungal genera including *Symmetrospora*, *Papiliotrema*, *Leucosporidium*, and *Holtermanniella* were uniquely identified in spoiled conventional DLGVJs. *Symmetrospora*, as the dominant fungal community in spoiled kale juice with the highest relative abundance, have been isolated from a wide diversity of leaf surfaces (Haelewaters et al., 2021; Li et al., 2020), It is also known to produce reddish pigments during growth, which can alter the color of fermenting products (Haelewaters et al., 2020). This observation aligns with the findings of this study, where kale juice showed the greatest changes in the a* (red-green) during storage. Interestingly, Bunbury-Blanchette et al. (2024) found that *Symmetrospora* were found only in North American hybrid wine grape grown organic vineyards, without presenting in conventional group, highlighting the microbial communities were highly impacted by the cropping system. *Papiliotrema*, another dominant fungal genus identified in spoiled collard greens juice, has been recognized as a core member of post-harvest fungal communities in broccoli (Kim and Park, 2021). It has been reported as a pectinolytic yeast capable of degrading pectin (Cadete et al., 2017), The pectinolytic enzymes produced by such yeasts can accelerate the softening of vegetable tissues, which may contribute to the loss of stability in DLGVJ products (Tournas, 2005). The spoilage mechanisms of core bacterial and fungal communities in both conventional and organic DLGVJs require more investigation in future studies.

## 5 Conclusion

This study characterized the changes of microbial communities in conventional DLGVJs during 21 days of refrigerated storage, contributing to a more comprehensive microbial profile by comparing them with their organic counterparts. Throughout storage, physicochemical properties, including pH and color were monitored in conventional chard, collard greens, and kale juices, revealing a general decrease in pH and more saturated color over time. Culture-dependent analysis revealed an overall decrease in aerobic plate counts (APC), while fungal populations exhibited an initial increase followed by a decline in conventional DLGVJs. High-throughput sequencing identified *Pseudomonas*, *Leuconostoc*, and *Periweissella* as core spoilage-associated bacterial genera, while *Sporobolomyces*, *Alternaria*, and *Symmetrospora* were recognized as predominant spoilage fungi in conventional DLGVJs. While there were notable similarities in microbial compositions between conventional and organic DLGVJs, distinct microbial communities were also observed, with some taxa uniquely associated with a specific cropping system. Based on the high relative abundance of lactic acid bacteria (LAB), particularly in spoiled kale and collard greens juices, a targeted culture-based approach was employed, resulting in the successful isolation and identification of two representative LAB species, *Enterococcus faecium* and *Leuconostoc mesenteroides*. These findings enhance our understanding of microbial ecology in DLGVJs and provide a scientific basis for developing targeted control strategies to maintain microbial quality and safety. One limitation associated with this study is that DLGVs used in this study were purchased from the same grocery store in the late summer and Fall 2024. Multiple trips were made to make sure different batches of the produce were used for the study. Future work incorporating broader geographic locations and storage temperatures is warranted to further confirm the involvement of LAB in the spoilage of DLGVJ.

## Data Availability

The datasets presented in this study can be found in online repositories. The names of the repository/repositories and accession number(s) can be found in the article/Supplementary material.
