# Supplementary material for: Identification and isolation of spoilage microbes in conventional dark leafy green vegetable juice during cold storage
Source: Front Microbiol. 2026 May 13;17:1831321. doi: 10.3389/fmicb.2026.1831321 (PMC13212437; doi:10.3389/fmicb.2026.1831321)
Supplement: Supplementary file 1 [file Data_Sheet_1.docx]

**Table S1** Pairwise comparison among conventional DLGVJs from on bacterial and fungal communities on Day 0.

| DLGVJs | Permanova (16S rRNA) |  | Permanova (ITS) |  |
| --- | --- | --- | --- | --- |
|  | F | *P*-value | F | *P*-value |
| Chard vs. Collard greens | 76.76 | 0.023* | 9.50 | 0.026* |
| Chard vs. Kale | 78.81 | 0.027* | 14.91 | 0.031* |
| Collard greens vs. Kale | 11.52 | 0.031* | 18.98 | 0.030* |

* *P*-value < 0.05 indicate the significant difference between two DLGVJs

**Table S2** Pairwise comparison among conventional DLGVJs from on bacterial and fungal communities on Day 21.

| DLGVJs | Permanova (16S rRNA) |  | Permanova (ITS) |  |
| --- | --- | --- | --- | --- |
|  | F | *P-*value | F | *P*-value |
| Chard vs. Collard greens | 222.78 | 0.022* | 27.80 | 0.033* |
| Chard vs. Kale | 94.78 | 0.028* | 17.33 | 0.030* |
| Collard greens vs. Kale | 0.35 | 0.91 | 4.77 | 0.031* |

* *P*-value < 0.05 indicate the significant difference between two DLGVJs
